# Efficient Data-Mining Algorithm for Predicting Heart Disease Based on an Angiographic Test

**DOI:** 10.21315/mjms2021.28.5.12

**Published:** 2021-10-26

**Authors:** Alabi Waheed Banjoko, Kawthar Opeyemi Abdulazeez

**Affiliations:** Department of Statistics, Faculty of Physical Sciences, University of Ilorin, Ilorin, Kwara State, Nigeria

**Keywords:** weighted support vector machine, biserial correlation, cross-validation, heart disease, splitting ratios

## Abstract

**Background:**

The computerised classification and prediction of heart disease can be useful for medical personnel for the purpose of fast diagnosis with accurate results. This study presents an efficient classification method for predicting heart disease using a data-mining algorithm.

**Methods:**

The algorithm utilises the weighted support vector machine method for efficient classification of heart disease based on a binary response that indicates the presence or absence of heart disease as the result of an angiographic test. The optimal values of the support vector machine and the Radial Basis Function kernel parameters for the heart disease classification were determined via a 10-fold cross-validation method. The heart disease data was partitioned into training and testing sets using different percentages of the splitting ratio. Each of the training sets was used in training the classification method while the predictive power of the method was evaluated on each of the test sets using the Monte-Carlo cross-validation resampling technique. The effect of different percentages of the splitting ratio on the method was also observed.

**Results:**

The misclassification error rate was used to compare the performance of the method with three selected machine learning methods and was observed that the proposed method performs best over others in all cases considered.

**Conclusion:**

Finally, the results illustrate that the classification algorithm presented can effectively predict the heart disease status of an individual based on the results of an angiographic test.

## Introduction

Heart disease is considered a life-threatening illness because the heart is a vital organ (1). The diagnosis of heart disease is usually based on symptoms, a physical assessment and a medical evaluation, such as the coronary angiographic test (2). Several different organisations have made recommendations regarding the optimal approach for identifying coronary heart disease in a patient in non-emergency settings (3). Several factors, such as smoking, level of cholesterol, obesity, hereditary issues and others, have been reportedly associated with heart disease (4). These factors, however, have different levels of association with heart disease and each is likely to be more pronounced in the angiographic diagnostic process of the patient.

The angiographic test is considered the gold standard for identifying and classifying a heart disease, such as coronary artery disease (5). However, there are side effects and complications associated with this test (6). Additionally, the associated risk of an angiogram has been attributed to cardiac and non-cardiac complications (5).

Early diagnosis of heart disease is paramount to its treatment (1), but medical practitioners typically face the challenge of timely detecting the presence or absence of heart disease, the kind of heart disease and the associated costs (7). Similarly, medical processes leading to diagnosis and predictions of some kinds of disease, such as cancer, have been reported to be quite inefficient due to the risks and time involved (8).

Automating the diagnostic process in health care services by using data-mining techniques is fast gaining recognition (9). Computerised classification of binary response data, such as the heart disease data used in this study, using data-mining algorithms can be useful for medical personnel to quickly diagnose their patients at a low cost (10). This approach will reduce the associated risks, costs, time and also proffer timely treatment and intervention (11).

This paper therefore presents an application of a modified support vector machine (SVM) called the weighted support vector machine (w-SVM) (11) method for the classification and prediction of heart disease. The w-SVM method was developed to improve the predictive power of the standard SVM (12) method using the biserial correlation between the response (presence or absence) of an angiographic test and the associated available factors on each patient.

## Methods

This section explains the data set and the methodology of the classification algorithm used in this study.

### Heart Disease Data Set

To assess the performance of the proposed w-SVM classifier, a secondary dataset on heart disease — which is available online and has been used in several works in the literature, such as Singh et al. and James et al. (4, 13) — was used in this research. The heart disease dataset is publicly available and can be obtained from the University of California Irvine (UCI) machine learning repository at http://archive.ics.uci.edu/ml/datasets/heart+Disease.

**Figure 1:**
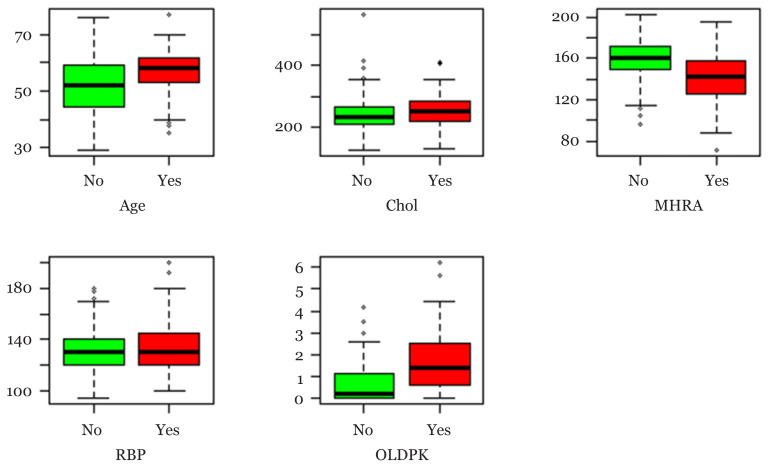
Box plot of the continuous variables in Table 3. The ‘No’ and ‘Yes’ indicates absence (−1) and presence (+1) of the heart disease, respectively

This study involved 303 patients that presented with chest pain. Out of these 303 heart disease patients, six subjects were excluded from the analysis due to incomplete information. This reduced the sample size to 297. The response variable (*y_i_* = ±1) is based on the result of the coronary angiographic test performed on each patient, with *y_i_* = 1 indicating the presence of heart disease and *y_i_* = −1 indicating the absence of heart disease. The data contained 76 attributes comprised of categorical factors and metrical covariates. In terms of the response category, as classified by the angiographic test, 137 (46.1%) of the 297 patients were classified as having heart disease while the remaining 160 (53.9%) did not have the disease. Most literature, such as Latha and Jeeva and, Suresh and Ananda Raj (14–15), recommend using only 14 out of the 75 attributes for prediction but after removing the missing data, there were 13 predictors on the subjects (13). These variables, and their respective categories, are presented in Table 1. All the predictor variables are labelled as *X*_1_, *X*_2_, ..., *X*_3_ respectively. Table 2 presents the frequency distribution of the presence or absence of the heart disease based on the angiographic result. Similarly, Table 3 and figure 1 presents some descriptive measures and box plots of the metrical covariate in the data respectively while Table 4 also presents the frequency and percentage distribution of the categorical factors in the data.

### Application of The Proposed Method on Heart Disease Data

The w-SVM algorithm assigns weights to the predictor variables in the data. As indicated in (11), the weights are first determined by obtaining the correlation between each of the predictor variables and the response variable. This is referred to as the point biserial correlation or association (16):


(i) $$r_{x_{j}Y}=\frac{{\bar{X}}_{+1}-{\bar{X}}_{-1}}{S_{X}}\sqrt{\frac{np_{+1}p_{-1}}{n-1}}$$

where *X̄*_+1_ and *X̄*_−1_ are the mean values of the predictor variable *X* for all data points in groups +1 and −1, respectively; *p*_+1_ and *p*_−1_ are the proportions of data points in groups +1 and −1, respectively; with py=nyn, *y* = −1, or +1, and in


(ii) $$S_{x}=\sqrt{\frac{1}{n-1}\sum_{i=1}^{n}{\left(X_{i}-\bar{X}\right)}^{2},}$$

the sample standard deviation of the *j^th^* feature *X_j_*.

These correlations determine the weight for each of the predictors. A step-by-step procedure of the w-SVM can be found in (11).

Suppose there are *n* sample points in the data with the *p* predictor variable *X*, and each point in *X* has an attribute in one of the binary classes, *y_i_* = ±1. The weight for each of the variables, as discussed in (11), is obtained as


(iii) $$\omega =\left(\begin{array}{cccc} \omega_{11} & \text{O} & \cdots & \text{O}\\ \text{O} & \omega_{22} & & \text{O}\\ : & : & \ddots & \text{O}\\ \text{O} & \text{O} & \cdots & \omega_{pp} \end{array}\right),$$

such that *tr* (*ω*) = 1.

Each weight *ω_ij_* in *ω* is computed by


(iv) $$\omega_{ij}=\frac{|r_{x_{j}Y}|}{\Sigma_{j=1}^{p}|r_{x_{j}Y}|}\;\text{for }j=1,2,\ldots ,p$$

where *r_x_*_*_j_*_*_Y_* is the correlation between predictor *X_j_* and the binary response *Y*.

Each of the weights are then multiplied by the respective predictor variables to give the new *n* × *p* data matrix *Z*. The traditional SVM is then applied on the new (weighted) data for the purpose of classification using the Monte-Carlo cross-validation (MCCV) method.

Different percentages of the splitting ratio were also considered for the train and test sets, respectively, as reported in (11). The quadratic programming problem of w-SVM is


(v) $$\underset{\alpha }{\text{min}}\;\left(\frac{1}{2}\sum_{i=1}^{n}\sum_{j=1}^{n}\alpha_{i}\alpha_{j}y_{i}y_{j}z_{i}z_{j}-\sum_{i=1}^{n}\alpha_{i}\right),$$

where *α_i_* ≥ 0 is the Lagrange multiplier.

As stated earlier, *Z* is the updated data, which was derived from the original data matrix (*X*) and the weight matrix, such that


(vi) $$Z_{n\times p}=X_{n\times p}\cdot \omega_{p\times p}$$

Therefore,


(vii) $$Z=\left(\begin{array}{cccc} Z_{11} & Z_{12} & \ldots & Z_{1p}\\ Z_{21} & Z_{22} & & Z_{2p}\\ : & : & \ddots & :\\ Z_{n1} & Z_{n2} & \ldots & Z_{np} \end{array}\right),$$

with *z_ij_* = *X_ij_ ω_jj_*, for *i* = 1, 2, …, *n* and *j* = 1, 2, …, *p*.

The data pre-processing and cleaning techniques have been applied to remove noisy and missing values present in the data set, respectively. The MCCV resampling technique is applied to produce balanced training and testing of the data set with different percentages of the splitting ratios and with replicating each split with 1,000 iterations. The w-SVM classification algorithm was developed to predict heart disease and the performance of the algorithm was validated with the test data. Figure 1 shows the overall flow of the prediction algorithm for heart disease.

The performance of the proposed w-SVM algorithm was evaluated via the test data using some performance indices. Given a 2 × 2 confusion matrix as presented in Table 5, where:

- True positive (TP) is the number of subjects that are positive and are predicted as such
- False positive (FP) is the number of subjects that are negative but predicted as positive
- False negative (FN) is the number of subjects that are positive but predicted as negative
- True negative (TN) is the number of subjects that are negative and are predicted as such, then

*N* = TP + FP + FN + TN is the total number of subjects in a test set.

Seven performance indices — Prediction accuracy (A_CC_), Misclassification error rate (MER), Sensitivity (S_e_), Specificity (S_p_), Positive predictive value (P_+_), Negative predictive value (P_−_) and Jaccard index (JI) — were used to assess the performance of the proposed method. The MER was used in comparing the w-SVM method with the three selected machine learning methods. A_CC_ is the proportion of subjects that are correctly classified by the classifier and it is defined as ${\text{A}}_{\text{cc}}=\frac{\text{TP}+\text{TN}}{\text{N}}$; MER is the proportion of subjects in the test set that are misclassified by the classifier/algorithm, defined as $\text{MFR}=\frac{\text{FP}+\text{FN}}{\text{N}}$; S_e_ is the proportion of true positive subjects that are correctly classified, defined as ${\text{S}}_{\text{e}}=\frac{\text{TP}}{\text{TP}+\text{FN}}$; S_p_ is the proportion of true negative subjects that are correctly classified, defined as ${\text{S}}_{\text{p}}=\frac{\text{TN}}{\text{FP}+\text{TN}}$; and P_+_ measures the precision of the classifier. This shows the proportion of the true class (*y* = 1) subjects that are correctly classified into that class among those that were classified as class 1 subjects by the classifier. It is defined as ${\text{P}}_{+}=\frac{\text{TP}}{\text{TP}+\text{FP}}$. Meanwhile, P_−_ is the proportion of group (*y* = −1) subjects that are correctly classified into that group among the subjects classified as group −1 subjects. It is defined as ${\text{P}}_{-}=\frac{\text{TN}}{\text{TN}+\text{FN}}$. Lastly, JI measures the similarity between the classifier and the subjects’ true class grouping, defined as $\text{JI}=\frac{\text{TP}}{\text{TP}+\text{FP}+\text{FN}}$.

All analyses were performed using R software (http://cran.r-project.org) version 3.4.4 with the e1071 package version 1.7-3.

## Results

This section presents the results of a heart disease classification using w-SVM.

### Biserial Correlation and Weight

The results in Table 6 show the degree of relationship between the binary response variable Y, and each of the predictor variables. The respective weight of the predictors as calculated using equation (ii) is presented in the third column of the table.

As a kernel-based learning method, the w-SVM uses different kernel functions in its classification process. The well-known kernel functions are linear, radial basis function (RBF), polynomial and sigmoid (12). The choice of kernel functions under different data structures has been extensively discussed in the literature (17). Based on the report of Banjoko et al. (17), the RBF kernel is adopted for the w-SVM algorithm on the heart disease data employed here. The optimal values of the w-SVM with RBF kernel parameters for the heart disease classification were determined via a 10-fold cross-validation method.

The weights presented in Table 6 are used to multiply each of the predictors before applying the traditional SVM for efficient classification using different percentages of the splitting ratio for both train and test data. It should be noted that the results in Table 7 are the values obtained from the test data which are not included in the algorithm training.

### Assessing the Efficiency of the Proposed Method on the Heart Disease Prediction

**Figure 2:**
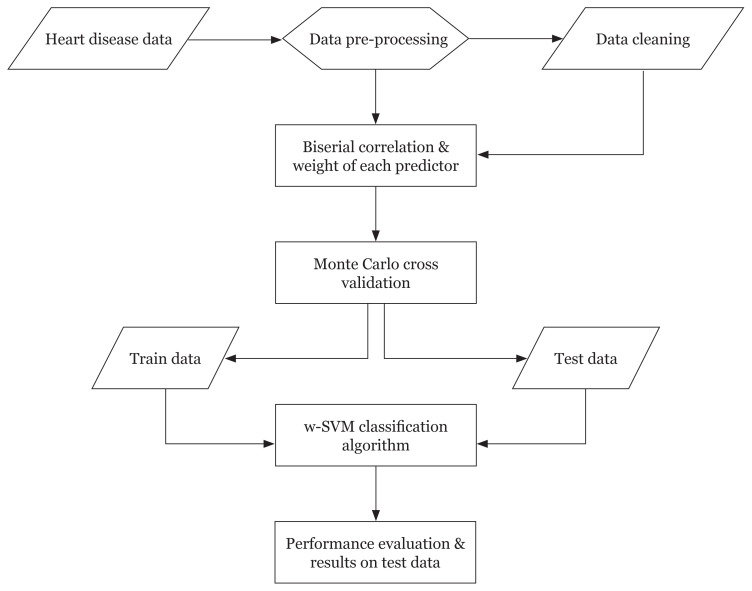
Flow chart of the w-SVM prediction algorithm for the heart disease data

The predictive performance of w-SVM and three other classification algorithms, namely Naïve Bayes (NB), Random Forest (RF) and SVM, are compared using the MER of the classifiers for the entire splitting ratio considered in this study. Worthy of note is the fact that the lower the MER, the better the classification result of such classifier. The results are presented in Figure 2.

## Discussion

This study has demonstrated an efficient data-mining method for the prediction of heart disease based on the results of an angiographic test. The step of the method of the w-SVM was able to identify the most and least correlated predictor variable with the response variable among the predictor variables considered in this study.

The results of the respective biserial correlations between the response variable and each of the predictor variables and the corresponding weights are presented in Table 6. Table 6 shows that the variable thalassemia (THAL) (*X*_13_) is the most correlated variable with the response variable having a correlation value of 0.5266 and corresponding weight value of 0.1347, while the predictor variable fasting blood sugar (FBS) (*X*_6_) is the least correlated variable with the response variable, with a correlation value of 0.0032 and a corresponding weight value of 0.0008.

The proposed method can unravel the importance of each of the predictor variables as they relate to heart disease. For instance, THAL has been reported in literature as a very important factor and even as indicative that the patient has a life-threatening heart disease (24). Similarly, FBS has been reported not to have a significant effect on heart disease (25). The above was rightly justified by the proposed algorithm through the weight of THAL and FBS, respectively, as demonstrated in Table 6.

Table 7 shows the several performance indices that were used to assess the performance of the w-SVM method on the heart disease data, using different percentages of the splitting ratio for the train and test sets. The results indicated that the splitting ratios considered in this study do not significantly affect the performance of w-SVM on the prediction of heart disease as the same results were achieved using different percentages of the splitting ratios. Generally, a rule of thumb in the data-mining technique is to split the data using the splitting ratio 80:20 for train and test data, respectively. Therefore, as observed in Table 6, the 80:20 splitting ratio gave a prediction accuracy of 90.53%, Sensitivity value of 90.99%, Specificity value of 90.72%, Positive predictive value of 87.99%, Negative predictive value of 90.53% and a Jaccard Index of 82.89%. The high value of the performance indices considered in the study testifies to the effectiveness of the w-SVM method in the prediction of heart disease. Throughout the different percentages of the splitting ratio considered, it was observed that the least prediction accuracy yielded of the w-SVM algorithm is 88.62% and was achieved at the splitting ratio 50:50. This splitting ratio is discouraged as it is usually very important to have more training sets than test sets in data-mining techniques. Similarly, the maximum accuracy is 90.60% and was achieved at the splitting ratio of 90:10. This is also not encouraged, because too small a percentage of the test set may lead to overfitting the classification model. A similar pattern of results was also observed for all other performance indices at different percentages of the splitting ratios.

The proposed method performs in a similar pattern for the different splitting ratios considered in this study. As pointed out earlier, the two extreme splitting ratios in this study (95:5 and 50:50) are discouraged. Therefore, it is advisable to use the rule of thumb of 80:20 for the train and test sets, respectively, in the implementation of the proposed algorithm.

**Figure 3:**
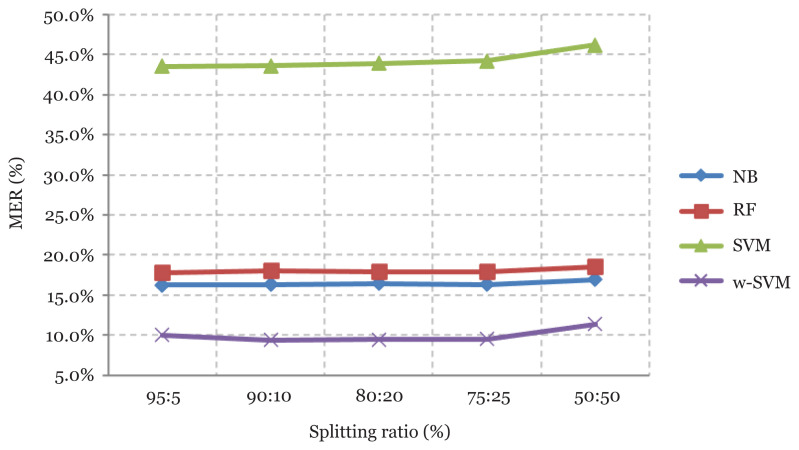
The graph of MER results of w-SVM, SVM, RF and NB

Also, the results presented in Figure 3 show the efficient performance of the w-SVM method over the selected three machine learning methods for the different splitting ratios using the MER of each of the methods considered in this study. Observations show that the w-SVM method performs best and is a more efficient data-mining technique — when compared to the three other existing classifiers — for predicting heart disease. This is indicated by the least MER values attained by the w-SVM throughout the splitting ratios considered.

The implication of the proposed method having the least MER is that, while the proposed method will be more efficient in correctly diagnosing a patient with a heart disease in this study, the other three selected methods will be less efficient because of the very high chance of wrongly diagnosing the patient. The other performance indices of the proposed method also justify how good is the proposed method.

The results of various performance indices for the classification of heart disease using the w-SVM as employed in this study is quite impressive and better when compared to the three selected existing classifiers. Similarly, the results obtained shows that the proposed method perform well as compared to some past studies using the same heart disease data set. Comparison of accuracy scores in the proposed method with the same Cleveland Heart Disease Data is presented in Table 8. It should be noted that the result of the proposed method is the average over 1,000 runs using MCCV — which make the results obtained more reliable — compared to some studies that have reported their results over a single run.

Finally, although the w-SVM seems to be effective in predicting heart disease by considering the relationship between the presence or otherwise of it and the associated factors (predictors), this study’s weakness is that, even as high as the prediction accuracy of the w-SVM is, there are still chances of misclassifying individuals with the same response to the heart disease when using the associated factors considered in this study. This is justified by the non-zero value of the MER over the different percentages of the splitting ratios. Therefore, there is a need to investigate the clinical importance of this study in order to further validate the results.

## Conclusion

This study presents an efficient data-mining algorithm for the classification and prediction of heart disease. The w-SVM algorithm was developed using the degree of association between the dichotomous response variable and each of the predictors to determine the respective weights of each of the predictor variables. The w-SVM provides better and more efficient results that will also assist domain experts with better planning of early diagnosis and treatment of the patient. The results show that the w-SVM algorithm can accurately predict heart disease based on an angiographic test with more than 90% accuracy. Medical practitioners, particularly cardiologists, are advised to further investigate the results of the proposed method for ease and quick medical treatment and intervention relating to heart disease based on an angiogram.

## Figures and Tables

**Table 1:** Description of the variables in the heart disease data

S/no	Factors	Description	Factor levels
1	Age	Patient age in years	Continuous
2	Sex	Patient sex	1 = male 0 = female
3	CP	Chest pain type	1 = typical angina 2 = atypical angina 3 = non-angina pain 4 = asymptomatic
4	RBP	Resting blood pressure	Continuous
5	Chol	Serum cholesterol in mg/dL	Continuous
6	FBS	Fasting blood sugar in mg/dL	1 ≥ 120 mg/dL 0 < 120 mg/dL
7	RECGR	Resting electrocardiographic results	0 = normal 1 = abnormality 2 = left ventricular hypertrophy
8	MHRA	Maximum heart rate achieved	Continuous
9	EXANG	Exercise-induced angina	0 = no 1 = yes
10	OLDPK	Depression induced by exercise relative to rest	Continuous
11	SLOPE	Slope of the peak exercise	1 = up sloping 2 = flat 3 = down sloping
12	CA	Number of major vessels	0 – 3 values
13	THAL	Defect type	3 = normal 6 = fixed 7 = reversible

**Table 2:** Angiographic test result

Heart disease absent (−)	Heart disease present (+)	Total
160 (53.9%)	137 (46.1%)	297 (100%)

**Table 3:** Clinical characteristics of the continuous variable for the entire 297 heart disease patient

	Minimum	Maximum	Mean (SD)
Age	29	77	55.5 (9.1)
Chol	126	564	247.4 (52.0)
MHRA	71	202	149.6 (22.9)
RBP	94	200	131.7 (17.8)
OLDPK	0	6.2	1.1 (1.2)

**Table 4:** Summary of the clinical characteristics of categorical variables for the entire 297 heart disease patient

Factors	Factor levels	Frequency (Percentage)
Total number of patient		297
Sex	Male	201 (67.7%)
	Female	96 (32.3%)
CP	Typical angina	23 (7.7%)
	Atypical angina	49 (16.5%)
	Non-angina pain	83 (27.9%)
	Asymptomatic	142 (47.8%)
FBS	≥120 mg/dL	254 (85.5%)
	< 120 mg/dL	43 (14.5%)
RECGR	Normal	147 (49.5%)
	Abnormality	4 (1.3%)
	Left ventricular hypertrophy	146 (49.2%)
EXANG	No	200 (67.3%)
	Yes	97 (32.7%)
SLOPE	Up sloping	139 (46.8%)
	Flat	137 (46.1%)
	Down sloping	21 (7.1%)
CA	0	174 (58.6%)
	1	65 (21.9%)
	2	38 (12.8%)
	3	20 (6.7%)
THAL	Normal	164 (55.2%)
	Fixed	18 (6.1%)
	Reversible	115 (38.7%)
NUM	Yes (Presence of heart disease)	137 (46.1%)
	No (Absence of heart disease)	160 (53.9%)

**Table 5:** A typical 2 × 2 confusion matrix for a binary response data

		True class (T)		
Predicted Class (P)		1	−1	Marginal total
	1	TP	FP	TP + FP
	−1	FN	TN	FN + TN
	Marginal total	TP + FN	FP + TN	N

**Table 6:** Degree of relationship and weight of each predictor

Variables in the data	*r* *_x_*_*_j_*_ *_Y_*	*ω* *_j_*	Rank
Age (*X*_1_)	0.2271	0.0581	9
Sex (*X*_2_)	0.2785	0.0712	8
CP (*X*_3_)	0.4089	0.1046	6
RBP (*X*_4_)	0.1535	0.0393	11
Chol (*X*_5_)	0.0803	0.0205	12
FBS (*X*_6_)	0.0032	0.0008	13
RECGR (*X*_7_)	0.1663	0.0425	10
MHRA (*X*_8_)	0.4238	0.1084	4
EXANG (*X*_9_)	0.4214	0.1078	5
OLDPK (*X*_10_)	0.4241	0.1085	3
SLOPE (*X*_11_)	0.3331	0.0852	7
CA (*X*_12_)	0.4632	0.1185	2
THAL (*X*_13_)	0.5266	0.1347	1

**Table 7:** Performance measures of w-WSM on the heart disease classification

Performance index (%)	Splitting ratios (%)				

	95:5	90:10	80:20	75:25	50:50
A_CC_	90.00	90.62	90.53	90.49	88.62
MER	10.00	9.38	9.47	9.51	11.38
S_e_	90.68	90.95	90.99	90.90	89.91
S_p_	90.34	91.02	90.72	90.78	88.50
P_+_	83.30	87.41	87.99	87.34	85.29
P_−_	89.95	90.54	90.53	90.53	89.82
JI	81.67	82.66	82.89	82.80	80.04

**Table 8:** Accuracy of Cleveland heart disease prediction with different

Authors	Year	Classifier used	Accuracy (%)
Otoom et al. (18)	2015	BayesNet	84.50
		SVM	85.10
		Functional trees	84.50
Vembandasamy et al. (19)	2015	Naïve Bayes	86.42
Dwivedi (20)	2018	Naïve Bayes	83.00
		Classification trees	77.00
		K-NN	80.00
		Logistic regression	85.00
		SVM	82.00
		ANN	84.00
Deepika et al. (21)	2016	Naïve Bayes	93.85
		Decision tree	92.59
		SVM	95.20
		ANN	94.27
Zriqat et al. (22)	2017	Decision tree	99.01
		Naïve Bayes	78.88
		Discriminant	83.50
		Random forest	93.40
		SVM	76.57
Rajdhan et al. (23)	2020	Decision tree	81.97
		Logistic regression	85.25
		Random forest	90.16
		Naïve Bayes	85.25
Proposed	2021	w-SVM	90.53

## References

[1] Capotosto L, Massoni F, De Sio S, Ricci S, Vitarelli A (2018). Early diagnosis of cardiovascular diseases in workers: role of standard and advanced echocardiography. Biomed Res Int.

[2] Abdissa SG, Deressa W, Shah AJ (2020). Incidence of heart failure among diabetic patients with ischemic heart disease: a cohort study. BMC Cardiovasc Disord.

[3] Chmiel C, Reich O, Signorell A, Tandjung R, Rosemann T, Senn O (2015). Appropriateness of diagnostic coronary angiography as a measure of cardiac ischemia testing in non-emergency patients — a retrospective cross-sectional analysis. PLoS One.

[4] Singh P, Singh S, Pandi-Jain GS (2018). Effective heart disease prediction system using data mining techniques. Int J Nanomedicine.

[5] Tavakol M, Ashraf S, Brener SJ (2012). Risks and complications of coronary angiography: a comprehensive review. Glob J Health Sci.

[6] Hessel SJ, Adams DF, Abrams HL (1981). Complications of angiography. Radiology.

[7] Amarnani A, Wengrofsky P, Tsui CL, Kariyanna PT, Kabani N, Salciccioli L (2020). Acute heart failure in scleroderma renal crisis: a case study for review of cardiac disease in systemic sclerosis. Am J Med Case Rep.

[8] Banjoko AW, Yahya WB, Garba MK (2019). Multiclass response feature selection and cancer tumour classification with support vector machine. Journal Biostat Epidemiol.

[9] Banjoko AW (2020). Data mining genome-based algorithm for optimal gene selection and prediction of colorectal carcinoma. Turkiye Klinikleri Journal of Biostatistics.

[10] Aziz R, Verma CK, Srivastava N (2016). A fuzzy based feature selection from independent component subspace for machine learning classification of microarray data. Genom Data.

[11] Banjoko AW, Yahya WB, Garba MK, Abdulazeez KO (2019). Weighted support vector machine algorithm for efficient classification and prediction of binary response data. J Phys Conf Ser.

[12] Vapnik VN (1995). The nature of statistical learning theory.

[13] James G, Witten D, Hastie T, Tibshirani R (2013). An introduction to statistical learning: with applications in R.

[14] Latha CBC, Jeeva SC (2019). Improving the accuracy of prediction of heart disease risk based on ensemble classification techniques. Informatics in Medicine Unlocked.

[15] Suresh P, Ananda Raj MD (2018). Study and analysis of prediction model for heart disease: an optimization approach using genetic algorithm. Int Jour Pure Appl Math.

[16] Gupta SD (1960). Point biserial correlation coefficient and its generalization. Psychometrika.

[17] Banjoko A, Yahya W, Garba M, Olaniran O, Dauda K, Olorede K (2015). Efficient support vector machine classification of diffuse large b-cell lymphoma and follicular lymphoma mRNA tissue samples. Annals Comput Sci Series.

[18] Otoom A, Abdallah E, Kilani Y, Kefaye A, Ashour M (2015). Effective diagnosis and monitoring of heart disease. Int J Softw Eng App.

[19] Vembandasamy K, Sasipriya R, Deepa E (2015). Heart diseases detection using Naive Bayes algorithm. Int J Innov Sci Eng Tech.

[20] Dwivedi AK (2018). Performance evaluation of different machine learning techniques for prediction of heart disease. Neural Comput Appl.

[21] Deepika K, Seema S (2016). Predictive analytics to prevent and control chronic diseases.

[22] Zriqat IA, Altamimi AM, Azzeh M (2016). A comparative study for predicting heart diseases using data mining classification methods. Int J Comp Sci Info Sec.

[23] Rajdhan A, Agarwal A, Sai M, Ghuli P (2020). Heart disease prediction using machine learning. Int J Eng Res Tech.

[24] Wood JC (2009). Cardiac complications in thalassemia major. Hemoglobin.

[25] Mongraw-Chaffin M, LaCroix AZ, Sears DD, Garcia L, Phillips LS, Salmoirago-Blotcher E (2017). A prospective study of low fasting glucose with cardiovascular disease events and all-cause mortality: the Women’s health initiative. Metabolism.

